# Influence of Silicate Concentrations on Growth, Carotenoid, and Fatty Acid Profiles of the Marine Diatom *Conticribra weissflogii*

**DOI:** 10.3390/md22110504

**Published:** 2024-11-06

**Authors:** David Kwame Amenorfenyo, Feng Li, Xiangyu Rui, Xianghu Huang, Changling Li

**Affiliations:** 1 College of Fisheries, Guangdong Ocean University, Zhanjiang 524088, China; davidamenorfenyo@yahoo.com (D.K.A.); 2112001098@stu.gdou.edu.cn (X.R.); huangxh@gdou.edu.cn (X.H.); licl@gdou.edu.cn (C.L.); 2 Guangdong Provincial Key Laboratory of Aquatic Animal Disease Control and Healthy Culture, Zhanjiang 524088, China

**Keywords:** *Conticribra weissflogii*, polyunsaturated fatty acids (PUFAs), fucoxanthin, carotenoids, fatty acids

## Abstract

Enhancing microalgal growth and bioactive compound production is becoming a duty for improving photosynthetic microorganisms. In this study, the growth, carotenoid, and fatty acid profiles of *Conticribra weissflogii* were studied under four different silicate concentrations and silicate-deficient conditions in an f/2 medium with continuous aeration, light intensity (30 ± 2 µmol m^−2^ s^−1^), salinity (25 ± 2‰), pH (8), and temperature (25 ± 2 °C). At the end of the experiment, we observed that a silicate concentration of 120 mg L^−1^ produced the maximum biomass dry weight (0.86 g L^−1^), carotenoid content (1.63 µg mL^−1^), and fucoxanthin content (1.23 mg g^−1^) in *C. weissflogii*. The eicosapentaenoic acid (EPA) (11,354.37 µg g^−1^), docosahexaenoic acid (DHA) (2516.16 µg g^−1^), gamma-linolenic acid (C18:3n6) (533.51 µg g^−1^), and arachidonic acid (C20:4n6) (1261.83 µg g^−1^) contents were significantly higher at Si 120 mg L^−1^. The results further showed the maximum fatty acid content in *C. weissflogii* at Si 120 mg L^−1^. However, the silicate-deficient conditions (Si 0 mg L^−1^) resulted in higher levels of saturated fatty acids (38,038.62 µg g^−1^). This study presents a practical approach for the large-scale optimization of biomass, carotenoid, fucoxanthin, and fatty acid production in *C. weissflogii* for commercial purposes.

## 1. Introduction

Diatoms are important primary producers in contemporary oceans and have emerged as major components of global biogenic nutrient cycles because of their ability to recycle carbon, silica, and sulfur [1]. They consist of more than 100,000 different species, making them the most diverse and abundant group of siliceous marine microorganisms [2,3,4]. Due to their complex evolutionary history, diatoms are capable of thriving in a range of environments, from polar regions to upwelling areas [4]. In addition to their ecological importance, the capacity of diatoms to biomineralize silica to create frustules makes them highly biocompatible for use as natural tools in drug delivery and as tailored micro- or nanodevices with a range of therapeutic applications [5]. Diatoms can vary significantly in size and morphology; however, they possess a distinctive outer cell wall (or frustule) composed of biogenic silica [6,7]. Diatoms have large storage vacuoles, allowing for greater nutrient storage within the cell and facilitating cell divisions, even in nutrient-depleted conditions, despite their large surface-to-volume ratio.

*Conticribra weissflogii*, previously classified as *Thalassiosira weissflogii*, is a diatom that is easily cultured and has gained extensive interest in the aquaculture and biotechnological industries because of its ability to biosynthesize fucoxanthin and PUFAs in considerable amounts [8,9]. Fucoxanthin, a unique carotenoid with distinctive antioxidant properties, has been shown to possess a wide range of health-promoting effects, including anti-obesity, anti-diabetic, and anti-cancer activities [10].

The production of fucoxanthin and polyunsaturated fatty acids (PUFAs) in microalgae is of considerable interest due to their significant health benefits and industrial applications [11,12]. *C. weissflogii*, a species of algae, has been studied for its potential to produce these valuable compounds. Although previous studies have focused on optimizing the growth conditions and nutrient concentrations to enhance the yield of fucoxanthin and PUFAs [9,13], the specific role of silicon in this process has not been extensively explored. Diatoms use silicon to construct their cell walls and play a role in the biogeochemical cycling of silicon in the ocean [14]. Silicate is a vital nutrient for the development and growth of diatoms, and its presence is crucial for defining the unique shapes of diatom species through their siliceous cell wall structures. However, a lack of silicate can impede cell growth, alter cell morphology, and affect lipid production in some specific diatom species [15,16]. The present study evaluates the influence of different silicate concentrations on the marine diatom *C. weissflogii* in terms of growth, carotenoid and fucoxanthin, and fatty acid production in *C. weissflogii*.

## 2. Results

### 2.1. Growth and Biomass Concentration of C. weissflogii

**Figure 1:**
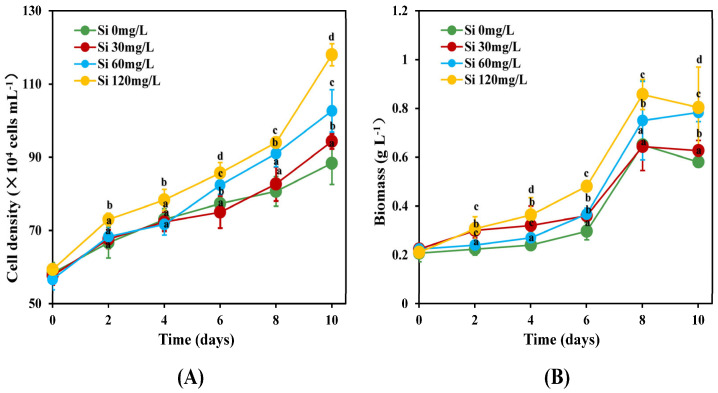
Changes in cell density (**A**) and biomass concentration (**B**) of *C. weissflogii* at silicate concentrations of 0, 30, 60, and 120 mg L^−1^ (mean ± SD, *n* = 3). Different alphabets indicate significant differences between groups (*p* < 0.05).

The growth of *C. weissflogii* at different silicate concentrations is shown in Figure 1. After 10 days of incubation, *C. weissflogii* demonstrated a higher cell density (Figure 1A) and biomass dry weight (Figure 1B) under high silicate concentrations (Si 120 mg L^−1^ and Si 60 mg L^−1^) compared to silicate concentrations of Si 0 mg L^−1^ and 30 mg L^−1^. The cell density of *C. weissflogii* under 0 mg L^−1^, 30 mg L^−1^, 60 mg L^−1^, and 120 mg L^−1^ increased throughout the incubation period. However, the biomass dry weight of *C. weissflogii* declined suddenly on day 10. Si 120 mg/L showed the maximum cell density (118 × 10^4^ cells mL^−1^) and dry weight (0.86 g L^−1^) on days 10 and 8, respectively, which were significantly (*p* < 0.05) higher compared to the other silicate concentration treatments.

### 2.2. Carotenoid and Fucoxanthin Contents of C. weissflogii

**Figure 2:**
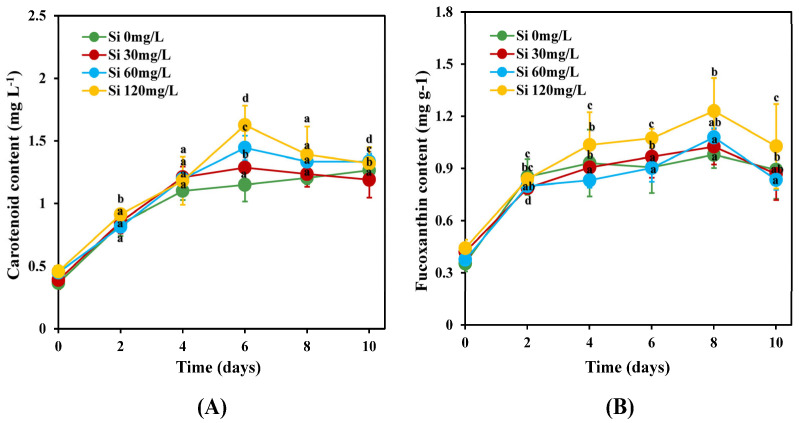
Changes in carotenoid (**A**) and fucoxanthin (**B**) content of *C. weissflogii* at different levels of silicate (mean ± SD, *n* = 3). Different alphabets indicate significant differences between groups (*p* < 0.05).

As shown in Figure 2A, with the exception of Si 0 mg L^−1^, the maximum carotenoid content of *C. weissflogii* in all the treatments (Si 30 mg L^−1^, Si 60 mg L^−1^, and Si 120 mg L^−1^) was obtained on day 6, with values of 1.30, 1.44, and 1.63 mg L^−1^ respectively. Si 0 mg L^−1^ had the lowest carotenoid content of 1.10 mg L^−1^ compared to the other silicate concentration treatments on day 6. However, the carotenoid content of *C. weissflogii* under Si 0 mg L^−1^ continued to increase until the final day of incubation, whereas the carotenoid content under Si 30 mg L^−1^ and Si 120 mg L^−1^ decreased on the final day of incubation. There was no significant difference (*p* < 0.05) in carotenoid content between all the treatments on the 4th and 8th days of incubation, but there were significant differences on the 2nd and 10th days.

Figure 2B shows the fucoxanthin content of *C. weissflogii* under various silicate concentrations (Si 0 mgL^−1^, Si 30 mg L^−1^, Si 60 mgL^−1^, and Si 120 mg L^−1^). With the exception of Si 0 mg L^−1^, the fucoxanthin content of *C. weissflogii* decreased only on day 10 in all the silicate treatment groups. However, there was a significant difference (*p* < 0.05) in fucoxanthin content between all silicate treatments on the 10th day of incubation. Figure 2B shows a decline in the fucoxanthin content of *C. weissflogii* under Si 0 mg L^−1^ on days 6 and 10, with values of 0.91 mg g^−1^ and 0.90 mg g^−1^, respectively. The results showed that the maximum fucoxanthin content in *C. weissflogii* at all silicate concentrations was obtained on day 8 of the incubation period. However, Si 120 mg L^−1^ obtained the highest fucoxanthin content, with a value of 1.23 mg g^−1^, compared to Si 60 mg L^−1^, Si 30 mg L^−1^, and Si 0 mg L^−1^, with values of 1.08, 1.03, and 0.98 mg g^−1^, respectively.

### 2.3. Fatty Acid Profile of C. weissflogii

At the end of the incubation period, the total fatty acid content of *C. weissflogii* was higher at Si 120 mg L^−1^ (74,508.02 µg g^−1^) compared to Si 0 mg L^−1^, Si 30 mg L^−1^, and Si 60 mg L^−1^ (Table 1). The eicosapentaenoic acid (EPA) (C20:5n3) and docosahexaenoic acid (DHA) (C22:6n3) contents were significantly (*p* < 0.05) higher at Si 120 mg L^−1^, with values of 11,354.37 and 2516.46 µg g^−1^, respectively. At 0 mg L^−1^, the saturated fatty acid content, such as C10:0, C11:0, C12:0, and C14:0, of *C. weissflogii* was significantly higher than that of the other silicate treatments. The results further showed that the gamma-linolenic acid (C18:3n6) and arachidonic acid (C20:4n6) contents were significantly (*p* < 0.05) higher at Si 120 mg L^−1^.

### 2.4. Biomass Productivity and Fucoxanthin Productivity of C. weissflogii

**Figure 3:**
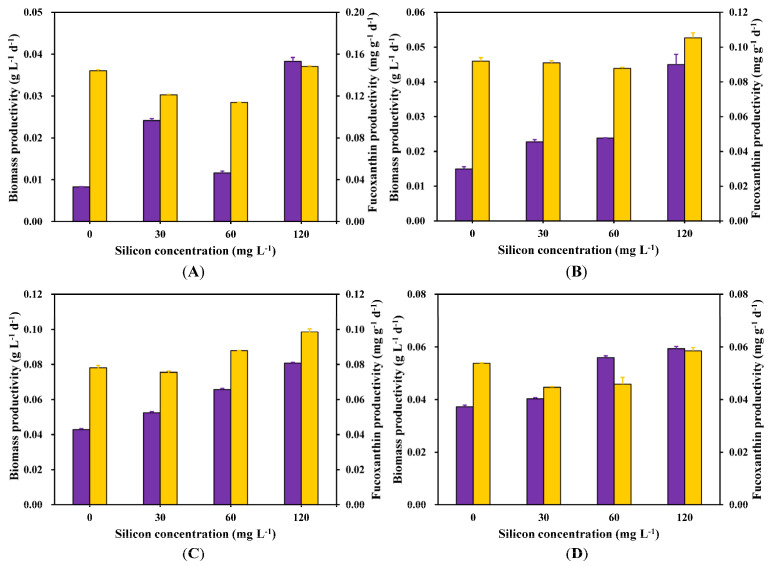
Biomass (purple) and fucoxanthin (yellow) productivity on day 4 (**A**), day 6 (**B**), day 8 (**C**), and day 10 (**D**). If the error bars are not visible, they are smaller than the symbol.

A comparative analysis of the biomass and fucoxanthin productivity was conducted on days 4, 6, 8, and 10 (Figure 3). *C. weissflogii* showed the maximum biomass and fucoxanthin productivity on the 8th day of the cultivation period for all the silicate treatments (Figure 3C). However, Si 120 mg L^−1^ produced the maximum biomass productivity (0.081 g L^−1^ d^−1^) during the same period.

## 3. Discussion

This study shows that silicate concentrations have an influence on growth, carotenoid and fucoxanthin, and fatty acid production in *C. weissflogii*.

Growth was induced over a wide range of silicate concentrations (Si 30 mg L^−1^ to Si 120 mg L^−1^). In this study, a silicate concentration of Si 0 mg L^−1^ led to a low peak in biomass dry weight and cell density on the 10th day of the incubation period. However, the lowest peaks for biomass dry weight (0.58 g L^−1^) and cell density (67 × 10^4^ cell m L^−1^) were observed on the 10th and 2nd days of incubation. These results were consistent with those of a previous study [17]. A similar result reported by [14] showed lower growth for the diatom *Nitzschia laevis* under Si 0 mg L^−1^. This phenomenon can be attributed to the absence of silicate in the growth medium. According to Umiatun et al. [18], a lack of silicate nutrients may cause improper frustule formation, an inability for cells to withstand osmotic pressure, and other environmental factors. There was a decline in the biomass dry weight of *C. weissflogii* under all the silicate treatments on the final day of cultivation, indicating that there may have been a decline in silicate in the culture medium towards the end of the cultivation period. Our study showed that silicate concentration had a significant (*p* < 0.05) effect on the growth and biomass production of *C. weissflogii.* Furthermore, this study showed that *C. weissflogii* can tolerate a silicate concentration of 120 mg L^−1^.

Photosynthesis is a process in which electromagnetic light energy is converted to chemically bound energy in the form of various organic compounds in phototropic cells and organisms. Pigments are chemical structures that play crucial roles in photosynthesis in microalgal cells. The distinctive pigment signature is not limited to photosynthetic pigments but also includes stress-related pigments, such as carotenoids. In marine ecosystems, fucoxanthin is a major carotenoid pigment, constituting about 10% of the total carotenoid content [19,20]. The results indicated that silicate concentration significantly (*p* < 0.05) affected carotenoid accumulation in *C. weissflogii*. As shown in Figure 2A, the carotenoid content was higher in the range of 30–120 mg L^−1^ for silicate concentrations. The maximum carotenoid content in *C. weissflogii* was achieved at Si 120 mg L^−1^ (1.63 µg mL^−1^). A study in [9] also showed that a higher carotenoid content (3.64 mg L^−1^) was achieved in *Chaetoceros* sp. at Si 120 mg L^−1^. In the current study, we observed that the carotenoid content of *C. weissflogii* increased as the silicate concentration increased. This could be attributed to the upregulation of photosynthetic efficiency due to the increased availability of silicon [21]. Currently, limited information is available on fucoxanthin biosynthesis in diatoms. Studies have shown that fucoxanthin accumulation is influenced by light intensity and is essential for the proper functioning of the light-harvesting complex in photosynthetic systems [22,23].

As illustrated in Figure 2B, fucoxanthin content was substantially enhanced by a sufficient level of silicate (Si 120 mg L^−1^). The highest content of fucoxanthin obtained was 1.23 mg g^−1^ at a silicate level of Si 120 mg L^−1^, followed by levels of Si 60 mg L^−1^ and Si 30 mg L^−1^, with values of 1.07 mg g^−1^ and 1.03 mg g^−1^, respectively. Our results (Figure 2B) were consistent with those reported by Mao et al. [24] and Yi et al. [25], who found that a high silicate concentration influenced the production of higher fucoxanthin contents in *N. laevis* and *P. tricornutum*, respectively. The present study further showed an observable decline in fucoxanthin content for all the treatments on the 10th day of incubation. This is likely due to a depletion of silicate concentration in the culture medium. Our findings were in agreement with those reported by Mao et al. [24], who found that depleted silicon concentration in a culture medium limits fucoxanthin accumulation in the marine diatom *Nitzschia laevis*. The optimal silicon concentration range for maximizing fucoxanthin content was between Si 60 mg L^−1^ and Si 120 mg L^−1^, which showed a positive correlation with biomass dry weight (Figure 1B). Furthermore, Si 120 mg L^−1^ led to the maximum fucoxanthin productivity (Figure 3) recorded on all the selected days, with the exception of day 2. This study showed that the accumulation of fucoxanthin in *C. weissflogii* was repressed by silicate deficiency. This was likely due to the association between fucoxanthin content and cell cycle progression.

Fatty acids (FAs) are the primary constituents of lipids within algal cells, serve as vital energy sources for algae during periods of stress, and play crucial roles in various physiological processes [9]. Si 120 mg L^−1^ resulted in the maximum TFA (Table 1) content, along with the maximum cell density and biomass dry weight (Figure 1). Valuable products, such as EPA and DHA, were significantly higher in parallel with biomass dry weight at high silicate concentrations (Si 120 mg L^−1^). FA is important for both human and animal nutrition because of its essential roles. [26,27,28]. These results were consistent with those of previous studies, which revealed that the EPA and DHA contents of *C. weissflogii* were 15.2% and 3.4%, respectively, under a silicate concentration of Si 120 mg/L compared with other silicate treatment groups. According to previous studies, the EPA and DHA contents of marine diatoms such as *S. costatum* and *C. calcitrans* range from 6% to 23.5% and 1.41 to 4% FA for *S. costatum* [29,30,31,32] and 5 to 26.3% and 0.7 to 2.3% FA for *C. calcitrans* [33,34,35,36,37], respectively. A positive correlation was observed between gamma-linolenic acid (C18:3n6), arachidonic acid (C20:4n6), and biomass dry weight. The C18:3n6 and C20:4n6 contents of *C. weissflogii* were significantly higher (*p* < 0.05) at Si 120 mg L^−1^. Linolenic acid can be converted to eicosapentaenoic acid and docosahexaenoic acid (DHA), which have been demonstrated to offer a range of protective benefits against various health conditions, including cardiovascular, neurological, osteoporotic, and inflammatory disorders [38].

## 4. Materials and Methods

### 4.1. Algal Strain and Culture Conditions

The microalgal strain, *C. weissflogii*, was obtained from the Algae Resource Development and Aquaculture Ecological Restoration Laboratory at Guangdong Ocean University, China. *C. weissflogii* was maintained in modified F/2 medium (NaNO_3_ (75 mg L^−1^), KH_2_PO_4_ (5 mg L^−1^), Na_2_SiO_3_-9H_2_O (20 mg L^−1^), F/2 trace element solution (1 mL), and F/2 vitamin solution (1 mL), with measurements given per liter of double-distilled water). The F/2 trace element solution comprised Na_2_EDTA·2H_2_O (4360 mg), FeC_6_H_5_O_7_ (3150 mg), MnCl_2_·4H_2_O (180 mg L^−1^), ZnSO_4_·4H_2_O (22 mg L^−1^), CuSO_4_·5H_2_O (9.8 mg L^−1^), Na_2_MoO_4_·2H_2_O (6.3 mg L^−1^), and CoCl_2_·6H_2_O (10 mg L^−1^), with measurements given per liter of distilled water. The F/2 vitamin solution was formulated with Biotin (0.5 mg L^−1^), Vitamin B_12_ (0.5 mg L^−1^), and Vitamin B_1_ (100 mg) (per liter of double-distilled water). The depleted f/2 culture medium was prepared by varying the initial concentrations of silicate, Na_2_SiO_3_·9H_2_O (Xilong Scientific Co., Ltd., Shantou, China; CAS No.: 13517-24-3, Analytical Reagent)

### 4.2. Experimental Setup

The cells from primary cultures were collected and concentrated by centrifugation (3000 rpm, 2 min), and the supernatant was removed. The collected cells were inoculated in a glass culture column (5 cm inner diameter, 60 cm high) in a Si-depleted f/2 culture medium (700 mL) with an inoculum density of about 6 × 10^5^ cells mL^−1^. Four Si concentration gradients were set at 0, 30, 60, and 120 mg L^−1^. The experiment was performed in triplicate under growth conditions depending on light intensity (30 ± 2 µmol m^−2^ s^−1^), salinity (25 ± 2‰), pH (8), and temperature (25 ± 2 °C) with a continuous supply of filtered air (aeration) for 10 days, as described in our previous study [39]. Samples were collected every other day to measure algal cell density, cell dry weight, carotenoid content, and fucoxanthin content.

### 4.3. Determination of Cell Density and Biomass Concentration

First, 5 mL samples were taken, and the cell density of *C. weissflogii* was determined using a Blood Counting Plate (25 mm × 16 mm) under an Olympus BX53 fluorescence microscope after the cells were stained with Lugol’s iodine solution [40]. Microalgal cells were centrifuged at 5000 rpm for 3 min and washed twice with distilled water. The sample was then filtered through pre-dried and pre-weighed (M_1_) filter paper (Whatman GF/C) and dehydrated overnight at 80 °C in a vacuum oven. The total mass was measured and recorded as M_2_. Dry weight (DW, g L^−1^) was determined using the following equation:

(1) $$DW=\left(M_{2}-M_{1}\right)\times 10^{3}/10$$

The biomass productivity (BP, g L^−1^ d^−1^) was calculated using the following equation:

(2) $$BP=\left({DW}_{t}-{DW}_{0}\right)/t$$

where DW_t_ and DW_0_ are the dry weights on day t and day 0, respectively.

### 4.4. Carotenoid and Fucoxanthin Extraction and Analysis

The carotenoid content of *C. weissflogii* was analyzed after extraction with 95% ethanol (*w*/*v*). Briefly, 10 mL of the suspension was filtered, freeze-dried at −20 °C for 12 h, and suspended in ethanol for 4 h in the dark. The suspensions were centrifuged at 5000 rpm for 10 min, and the pigment content of the supernatant was spectrophotometrically measured at 480, 510, and 750 nm. The carotenoid content was calculated using a previously described equation [41].

(3) $$P\left(Carotenoids\right)=7.6\times \left[\left(A_{480}-A_{750}\right)-1.49\times \left(A_{510}-A_{750}\right)\right]$$

where *A* is the absorbance.

Fucoxanthin was extracted as previously described [9]. *C. weissflogii* (80 mL) was centrifuged at 5000 rpm for 10 min at 4 °C, and the biomass was freeze-dried for 2 days. The freeze-dried biomass was grinded into powder, added to absolute ethanol (1 g:40 mL), and extracted twice for 1 h at 60 °C in the dark. After extraction, the algal solution was centrifuged at 5000 rpm for 10 min, and the supernatant was collected and measured using an ultraviolet spectrophotometer. The optical density of the supernatant was determined at 445 nm (D_445_), and the fucoxanthin content was calculated according to the following formula [42]:

(4) $$C=\left(1000\times A_{445}\times N\times V\right)/\left(A^{\prime }\times M\times 100\right)$$

where *C* is the fucoxanthin content (mg g^−1^), *D*_445_ is the optical density of fucoxanthin at 445 nm, *N* is the dilution factor, *V* is the volume of the crude extract, *A*′ is the theoretical absorption value of the solute with a volume fraction of 1% in a cuvette with a long path length of 1 cm (1600), and *M* is the mass of the sample to be measured [39].

The fucoxanthin productivity (FP, mg g^−1^ d^−1^) was calculated using the following equation:

(5) $$FP=\left(C_{t}-C_{0}\right)/t$$

where C_t_ and C_0_ are the fucoxanthin contents on day t and day 0, respectively.

### 4.5. FAME Test and GC Analysis

Fatty acids were converted to fatty acid methyl esters (FAMEs) according to a method described in a previous study [43]. Specifically, 0.1 g of wet microalgal biomass was hydrolyzed and then methyl-esterified with 2 mL of acetyl chloride (100%) in 20 mL of methanol solution at 90 °C while stirring at 500 rpm. The samples were then filtered using 90 mm Whatman filter paper and washed with 10 mL of methanol after direct transesterification. A rotary evaporator (IKA RV 10 digital; Guangzhou, China) was used to evaporate methanol, and the sample was manually shaken for 5 min after the addition of hexane (10 mL). Hexane was evaporated to remove the hexane layer. The recovered FAMEs were analyzed using a Perkin Elmer Clarus 680 gas chromatograph (GC) equipped with a Perkin Elmer Clarus 680 flame ionization detector (FID) (PerkinElmer; Shelton, CT, USA) and Thermo Scientific TG-Polar column, (Thermo Scientific; Shanghai, China. The oven temperature was initially set to 50 °C for 5 min, raised to 260 °C at a rate of 7 °C min^−1^, and maintained at 260 °C for 5 min. The temperature of the injector and detector was 260 °C, and helium was used as the carrier gas. The FAME peaks in the samples were identified by comparing their retention times with those of the standards (Supelco TM 37 component FAME mix, Sigma-Aldrich, St. Louis, MI, USA). Data processing: fatty acid components were identified by comparing retention times with fatty acid methyl ester standards and quantified using the area normalization method.

The content of each fatty acid in the sample is calculated according to Equation (6):

(6) $$X_{i}=\frac{A_{i}\times m_{s_{i}}\times F_{TG_{i}-{FA}_{i}}}{A_{S_{i}}\times m}\times 100$$

where *X_i_* (g/100 g) is the content of each fatty acid in the sample; A_i_ is the peak area of each fatty acid methyl ester in the sample measurement solution; $m_{s_{i}}$ (mg) is the quality of the standards contained in the standard working solution with the fatty acid triglyceride absorbed; $F_{{FG}_{i}-{FA}_{i}}$ is the conversion coefficient for the conversion of each fatty acid triglyceride to fatty acid; $A_{s_{i}}$ is the peak area of each fatty acid in the standard measuring solution; m (mg) is the weighing quality of the sample; and 100 is a coefficient that converts the content to the content per 100 g of sample.

### 4.6. Statistical Analysis

One-way ANOVA with Duncan’s test (post hoc) was used to conduct statistical analyses using the SPSS for Windows statistical software package (IBM SPSS v26.0; San Diego, CA, USA). The significance level was set at *p* < 0.05, and the results are expressed as the mean ± SD.

## 5. Conclusions

In this study, the effects of different silicate concentrations (0–120 mg L^−1^) on the cell growth, carotenoid and fucoxanthin synthesis, and fatty acid profiles of *C. weissflogii* were studied. These results revealed that *C. weissflogii* can tolerate silicon concentrations ranging up to 120 mg L^−1^, which can effectively promote cell growth and biomass production, as well as the production of carotenoids, fucoxanthins, and fatty acids. The impact of silicate concentration on the production of fucoxanthin, EPA, and DHA, as well as other valuable products, in *C. weissflogii* represents an intriguing area of research with implications in the aquaculture, food, and pharmaceutical industries.

## Figures and Tables

**Table 1:** Fatty acid composition and content (µg g^−1^) of *C. weissflogii* at silicate concentrations of 0, 30, 60, and 120 mg L^−1^ at the end of the culture period. Values (mean ± SD; *n* = 3) in the same row having a common superscript are not significantly different (*p* < 0.05).

Fatty Acids	0 mg/L	30 mg/L	60 mg/L	120 mg/L
C8:0	61.19 ± 0.01	57.31 ± 0.24	Not detected	Not detected
C10:0	329.16 ± 0.27 ^d^	36.28 ± 0.27 ^a^	113.25 ± 0.26 ^c^	51.34 ± 0.28 ^b^
C11:0	171.59 ± 0.14 ^d^	162.44 ± 0.03 ^c^	125.16 ± 0.44 ^b^	114.85 ± 0.15 ^a^
C12:0	103.60 ± 0.53 ^d^	84.76 ± 0.02 ^c^	51.15 ± 0.24 ^b^	33.04 ± 0.44 ^a^
C14:0	8101.76 ± 0.32 ^d^	7850.14 ± 0.50 ^c^	1047.63 ± 0.36 ^a^	7479.86 ± 0.61 ^b^
C14:1n5	177.12 ± 0.31 ^c^	153.97 ± 0.33 ^b^	Not detected	134.68 ± 0.60 ^a^
C15:0	1244.84 ± 0.53 ^c^	1040.50 ± 1.05 ^b^	149.05 ± 0.38 ^a^	1378.94 ± 0.78 ^d^
C16:0	25,928.18 ± 0.2 ^c^	22,521.40 ± 0.80 ^b^	28,236.66 ± 1.37 ^d^	20,984.98 ± 0.19 ^a^
C16:1n7	20,985.50 ± 1.57 ^a^	26,213.11 ± 1.07 ^c^	30,546.97 ± 0.38 ^d^	24,523.56 ± 1.03 ^b^
C18:0	456.18 ± 0.59 ^a^	546.27 ± 0.76 ^d^	436.51 ± 0.82 ^a^	470.26 ± 0.64 ^c^
C18:1n9c	1093.79 ± 1.01 ^d^	730.35 ± 0.75 ^c^	63.73 ± 0.56 ^a^	643.58 ± 1.20 ^b^
C18:2n6c	366.31 ± 1.50 ^a^	778.82 ± 0.70 ^c^	556.34 ± 1.40 ^b^	953.53 ± 1.0 ^d^
C18:3n6	115.56 ± 0.11 ^a^	390.11 ± 0.64 ^c^	362.94 ± 0.59 ^b^	533.51 ± 0.45 ^d^
C18:3n3	208.35 ± 0.21 ^c^	155.63 ± 0.45 ^b^	35.46 ± 0.47 ^a^	221.29 ± 1.02 ^d^
C20:0	16.48 ± 0.89 ^c^	11.50 ± 0.34 ^b^	11.95 ± 0.45 ^a^	84.37 ± 0.58 ^d^
C20:2	155.92 ± 0.21 ^d^	39.98 ± 0.36 ^b^	24.93 ± 0.34 ^a^	89.82 ± 0.59 ^c^
C20:3n6	31.78 ± 0.64 ^a^	94.93 ± 0.53 ^c^	76.19 ± 0.35 ^b^	155.17 ± 0.68 ^d^
C20:4n6	221.13 ± 1.20 ^b^	982.72 ± 0.22 ^c^	22.74 ± 1.04 ^a^	1261.83 ± 0.21 ^d^
C20:3n3	191.68 ± 0.11 ^d^	154.65 ± 0.05 ^c^	23.02 ± 0.58 ^a^	80.04 ± 0.15 ^b^
C20:5n3	8121.98 ± 0.20 ^c^	7903.84 ± 0.23 ^b^	6944.49 ± 0.85 ^a^	11,354.37 ± 0.21 ^d^
C22:0	149.10 ± 0.1 ^b^	169.58 ± 0.74 ^d^	31.93 ± 0.26 ^a^	156.16 ± 0.24 ^c^
C22:6n3	2124.03 ± 0.24 ^c^	1767.99 ± 0.17 ^a^	1995.96 ± 0.57 ^b^	2516.46 ± 0.30 ^d^
C23:0	332.63 ± 0.49 ^c^	242.86 ± 0.30 ^a^	261.96 ± 0.12 ^b^	376.97 ± 0.25 ^d^
C24:0	811.28 ± 0.78 ^c^	786.57 ± 0.48 ^b^	680.98 ± 0.28 ^a^	907.18 ± 0.37 ^d^
SFA	38,038.62	33,752.47	31,408.19	32,414.92
UFA	34,249.33	39,912.37	41,089.28	42,938.10
TFA	72,288.95	73,664.84	71,797.47	74,508.02

Note: SFA; saturated fatty acid; UFA; unsaturated fatty acid; TFA; total fatty acid.

## Data Availability

The original data presented in the study are included in the article; further inquiries can be directed to the corresponding author.

## References

[1] Kranzler C.F., Krause J.W., Brzezinski M.A., Edwards B.R., Biggs W.P., Maniscalco M., McCrow J.P., Van Mooy B.A.S., Bidle K.D., Allen A.E. (2019). Silicon limitation facilitates virus infection and mortality of marine diatoms. Nat. Microbiol..

[2] Fu W., Shu Y., Yi Z., Su Y., Pan Y., Zhang F., Brynjolfsson S. (2022). Diatom morphology and adaptation: Current progress and potentials for sustainable development. Sustain. Horiz..

[3] Mann D.G., Vanormelingen P. (2013). An inordinate fondness? the number, distributions, and origins of diatom species. J. Eukaryot. Microbiol..

[4] Malviya S., Scalco E., Audic S., Vincent F., Veluchamy A., Poulain J., Wincker P., Iudicone D., De Vargas C., Bittner L. (2016). Insights into global diatom distribution and diversity in the world’s ocean. Proc. Natl. Acad. Sci. USA.

[5] Delasoie J., Zobi F. (2019). Natural diatom biosilica as microshuttles in drug delivery systems. Pharmaceutics.

[6] Zhang S., Liu H., Ke Y., Li B. (2017). Effect of the silica content of diatoms on protozoan grazing. Front. Mar. Sci..

[7] Martin-Jézéquel V., Hildebrand M., Brzezinski M.A. (2000). Silicon metabolism in diatoms: Implications for growth. J. Phycol..

[8] Marella T.K., Tiwari A. (2020). Marine diatom *Thalassiosira weissflogii* based biorefinery for co-production of eicosapentaenoic acid and fucoxanthin. Bioresour. Technol..

[9] Rui X., Amenorfenyo D.K., Peng K., Li H., Wang L., Huang X., Li C., Li F. (2023). Effects of different nitrogen concentrations on co-production of fucoxanthin and fatty acids in *Conticribra weissflogii*. Mar. Drugs.

[10] Kumarasinghe H.S., Gunathilaka M.D.T.L. (2024). A systematic review of fucoxanthin as a promising bioactive compound in drug development. Phytochem. Lett..

[11] Guihéneuf F., Stengel D.B. (2013). LC-PUFA-enriched oil production by microalgae: Accumulation of lipid and triacylglycerols containing n-3 LC-PUFA is triggered by nitrogen limitation and inorganic carbon availability in the marine haptophyte *Pavlova* lutheri. Mar. Drugs.

[12] Kanamoto A., Kato Y., Yoshida E., Hasunuma T., Kondo A. (2021). Development of a method for fucoxanthin production using the haptophyte marine microalga *Pavlova* sp. OPMS 30543. Mar. Biotechnol..

[13] Peng K., Amenorfenyo D.K., Rui X., Huang X., Li C., Li F. (2024). Effect of iron concentration on the co-production of fucoxanthin and fatty acids in *Conticribra weissflogii*. Mar. Drugs.

[14] Benoiston A.S., Ibarbalz F.M., Bittner L., Guidi L., Jahn O., Dutkiewicz S., Bowler C. (2017). The evolution of diatoms and their biogeochemical functions. Philos. Trans. R. Soc. B Biol. Sci..

[15] Jiang Y., Laverty K.S., Brown J., Brown L., Chagoya J., Burow M., Quigg A. (2015). Effect of silicate limitation on growth, cell composition, and lipid production of three native diatoms to Southwest Texas desert. J. Appl. Phycol..

[16] Yamada K., Yoshikawa S., Ichinomiya M., Kuwata A., Kamiya M., Ohki K. (2014). Effects of silicon-limitation on growth and morphology of *Triparma laevis* NIES-2565 (Parmales, Heterokontophyta). PLoS ONE.

[17] Silviananda A., Hadi E. (2024). The influence of differences in silicate concentration on the growth of microalgae *Thalassiosira* sp. at the laboratory scale. J. Mar. Biotechnol. Immunol..

[18] Umiatun S., Carmudi C., Christiani C. (2017). Hubungan antara kandungan silika dengan kelimpahan diatoma benthik di sepanjang sungai pelus kabupaten banyumas (The relationship between silica content and benthic diatom abundance along the Pelus River, Banyumas Regency). Scr. Biol..

[19] Krinsky N.I., Johnson E.J. (2005). Carotenoid actions and their relation to health and disease. Mol. Asp. Med..

[20] Muthuirulappan S., Francis S.P. (2013). Anti-cancer mechanism and possibility of nano-suspension formulation for a marine algae product fucoxanthin. Asian Pac. J. Cancer Prev..

[21] Kumar Singh P., Bhattacharjya R., Kiran Marella T., Saxena A., Mishra B., Savio S., Congestri R., Sindhu R., Binod P., Tiwari A. (2022). Production of lipids and proteins from marine diatoms under changing pH and silica. Bioresour. Technol..

[22] Bertrand M. (2010). Carotenoid biosynthesis in diatoms. Photosynth. Res..

[23] Gómez-Loredo A., Benavides J., Rito-Palomares M. (2016). Growth kinetics and fucoxanthin production of *Phaeodactylum tricornutum* and *Isochrysis galbana* cultures at different light and agitation conditions. J. Appl. Phycol..

[24] Mao X., Chen S.H.Y., Lu X., Yu J., Liu B. (2020). High silicate concentration facilitates fucoxanthin and eicosapentaenoic acid (EPA) production under heterotrophic condition in the marine diatom *Nitzschia laevis*. Algal Res..

[25] Yi Z., Su Y., Cherek P., Nelson D.R., Lin J., Rolfsson O., Wu H., Salehi-Ashtiani K., Brynjolfsson S., Fu W. (2019). Combined artificial high-silicate medium and LED illumination promote carotenoid accumulation in the marine diatom *Phaeodactylum tricornutum*. Microb. Cell Fact..

[26] Remize M., Brunel Y., Silva J.L., Berthon J.Y., Filaire E. (2021). Microalgae n-3 PUFAs production and use in food and feed industries. Mar. Drugs.

[27] Bastos C.R.V., Maia I.B., Pereira H., Navalho J., Varela J.C.S. (2022). Optimisation of biomass production and nutritional value of two marine diatoms (Bacillariophyceae), *Skeletonema costatum* and *Chaetoceros calcitrans*. Biology.

[28] Pereira H., Barreira L., Figueiredo F., Custódio L., Vizetto-Duarte C., Polo C., Rešek E., Aschwin E., Varela J. (2012). Polyunsaturated fatty acids of marine macroalgae: Potential for nutritional and pharmaceutical applications. Mar. Drugs.

[29] Guihéneuf F., Mimouni V., Ulmann L., Tremblin G. (2008). Environmental factors affecting growth and omega 3 fatty acid composition in skeletonema costatum. the influences of irradiance and carbon source. Diatom Res..

[30] Pennarun A.L., Prost C., Haure J., Demaimay M. (2003). Comparison of two microalgal diets. 1. Influence on the biochemical and fatty acid compositions of raw oysters (*Crassostrea gigas*). J. Agric. Food Chem..

[31] Houcke J., Medina I., Maehre H.K., Cornet J., Cardinal M., Linssen J., Luten J. (2017). The effect of algae diets (*Skeletonema costatum* and *Rhodomonas baltica*) on the biochemical composition and sensory characteristics of Pacific cupped oysters (*Crassostrea gigas*) during land-based refinement. Food Res. Int..

[32] Gao G., Wu M., Fu Q., Li X., Xu J. (2019). A two-stage model with nitrogen and silicon limitation enhances lipid productivity and biodiesel features of the marine bloom-forming diatom *Skeletonema costatum*. Bioresour. Technol..

[33] Delaunay F., Marty Y., Moal J., Samain J.F. (1993). The effect of monospecific algal diets on growth and fatty acid composition of *Pecten maximus* (L.) larvae. J. Exp. Mar. Bio. Ecol..

[34] Volkman J.K., Jeffrey S.W., Nichols P.D., Rogers G.I., Garland C.D. (1989). Fatty acid and lipid composition of 10 species of microalgae used in mariculture. J. Exp. Mar. Bio. Ecol..

[35] Fernández-Reiriz M.J., Perez-Camacho A., Ferreiro M.J., Blanco J., Planas M., Campos M.J., Labarta U. (1989). Biomass production and variation in the biochemical profile (total protein, carbohydrates, RNA, lipids and fatty acids) of seven species of marine microalgae. Aquaculture.

[36] Rivero-Rodríguez S., Beaumont A.R., Lora-Vilchis M.C. (2007). The effect of microalgal diets on growth, biochemical composition, and fatty acid profile of *Crassostrea corteziensis* (Hertlein) juveniles. Aquaculture.

[37] Kaspar H.F., Keys E.F., King N., Smith K.F., Kesarcodi-Watson A., Miller M.R. (2014). Continuous production of *Chaetoceros calcitrans* in a system suitable for commercial hatcheries. Aquaculture.

[38] Barve K.H., Kulkarni Y.A., Gaikwad A.B. (2016). Nutraceuticals as therapeutic agents for inflammation. Fruits, Vegetables, and Herbs: Bioactive Foods in Health Promotion.

[39] Pan Y., Amenorfenyo D.K., Dong M., Zhang N., Huang X., Li C., Li F. (2024). Effects of salinity on the growth, physiological and biochemical components of microalga *Euchlorocystis marina*. Front. Mar. Sci..

[40] Li F., Rui X., Amenorfenyo D.K., Pan Y., Huang X., Li C. (2023). Effects of temperature, light and salt on the production of fucoxanthin from *Conticribra weissflogii*. Mar. Drugs.

[41] Parsons T.R., Maita Y., Lalli C.M. (1984). Determination of chlorophylls and total carotenoids: Spectrophotometric method—Science Direct. A Manual of Chemical & Biological Methods for Seawater Analysis.

[42] Xu R., Gong Y., Cheng W., Li S., Chen R., Zheng X., Cheng X., Wang H. (2019). Effects of LED monochromatic light quality of different colors on fucoxanthin content and expression levels of related genes in *Phaeodactylum Tricornutum*. Acta Opt. Sin..

[43] Ge S., Qiu S., Tremblay D., Viner K., Champagne P., Jessop P.G. (2018). Centrate wastewater treatment with *Chlorella vulgaris*: Simultaneous enhancement of nutrient removal, biomass and lipid production. Chem. Eng. J..

